# Omega-3 Polyunsaturated Fatty Acids Inhibit the Function of Human URAT1, a Renal Urate Re-absorber

**DOI:** 10.3390/nu12061601

**Published:** 2020-05-29

**Authors:** Hiroki Saito, Yu Toyoda, Tappei Takada, Hiroshi Hirata, Ami Ota-Kontani, Hiroshi Miyata, Naoyuki Kobayashi, Youichi Tsuchiya, Hiroshi Suzuki

**Affiliations:** 1 Frontier Laboratories for Value Creation, Sapporo Holdings Ltd., 10 Okatome, Yaizu, Shizuoka 425-0013, Japan; hiroki.saito@sapporoholdings.co.jp (H.S.); hiroshi.hirata@sapporoholdings.co.jp (H.H.); ami.ota@sapporoholdings.co.jp (A.O.-K.); naoyuki.kobayashi@sapporoholdings.co.jp (N.K.); yoichi.tsuchiya@sapporoholdings.co.jp (Y.T.); 2 Department of Pharmacy, The University of Tokyo Hospital, 7-3-1 Hongo, Bunkyo-ku, Tokyo 113-8655, Japan; ytoyoda-tky@umin.ac.jp (Y.T.); hmiyata-tky@umin.ac.jp (H.M.); suzukihi-tky@umin.ac.jp (H.S.)

**Keywords:** Docosahexaenoic acid, eicosapentaenoic acid, functional food, gout, human health, hyperuricemia, PUFA, SLC22A12, transporter, uric acid, uricosuric activity

## Abstract

The beneficial effects of fatty acids (FAs) on human health have attracted widespread interest. However, little is known about the impact of FAs on the handling of urate, the end-product of human purine metabolism, in the body. Increased serum urate levels occur in hyperuricemia, a disease that can lead to gout. In humans, urate filtered by the glomerulus of the kidney is majorly re-absorbed from primary urine into the blood via the urate transporter 1 (URAT1)-mediated pathway. URAT1 inhibition, thus, contributes to decreasing serum urate concentration by increasing net renal urate excretion. Here, we investigated the URAT1-inhibitory effects of 25 FAs that are commonly contained in foods or produced in the body. For this purpose, we conducted an in vitro transport assay using cells transiently expressing URAT1. Our results showed that unsaturated FAs, especially long-chain unsaturated FAs, inhibited URAT1 more strongly than saturated FAs. Among the tested unsaturated FAs, eicosapentaenoic acid, α-linolenic acid, and docosahexaenoic acid exhibited substantial URAT1-inhibitory activities, with half maximal inhibitory concentration values of 6.0, 14.2, and 15.2 μM, respectively. Although further studies are required to investigate whether the ω-3 polyunsaturated FAs can be employed as uricosuric agents, our findings further confirm FAs as nutritionally important substances influencing human health.

## 1. Introduction

Fatty acids (FAs) are physiologically important as energy sources and membrane constituents; moreover, FAs have diverse biological activities that modulate numerous cell/tissue properties in living organisms [1]. Accumulating evidence suggests that via such actions, dietary FAs can influence human health, well-being, and the risk of disease development. For instance, intake of polyunsaturated fatty acids (PUFAs) of the ω-3 family, such as eicosapentaenoic acid (EPA) and docosahexaenoic acid (DHA), has been considered to reduce the risk of chronic diseases including cardiovascular diseases; however, other effects of FAs consumption on human health remain controversial and need further investigation [2,3,4,5]. In addition to cardiovascular diseases, it is currently acknowledged that FAs influence a range of other diseases, such as metabolic and inflammatory diseases, as well as cancer [1]. However, little is known about the association between FAs and hyperuricemia, a common urate-related disease, especially regarding the effects of FAs on urate-handling machineries in the body.

Hyperuricemia, which is characterized by elevated serum uric acid (SUA) levels, is a lifestyle-related disease with high prevalence [6]. As hyperuricemia is a risk factor for gout, a very common form of inflammatory arthritis, SUA management at appropriate levels is becoming increasingly important in daily life [7,8]. Due to the lack of functional uricase (urate-degrading enzyme) in humans [9], uric acid is the end-product of human purine metabolism; urate excretion from the body is, therefore, necessary for the maintenance of uric acid homeostasis. The kidney is responsible for elimination of approximately two-thirds of urate [10]. However, only 3%–10% of the urate filtered by the glomerulus of the kidney is secreted to the urine [11] because most of it is re-absorbed from the primary urine into the blood in the proximal tubule by the urate transporter 1 (URAT1, also known as SLC22A12)-mediated pathway [12]. Thus, inhibition of this urate re-absorption pathway contributes to SUA lowering via the increase of net renal urate excretion.

URAT1 is a physiologically important renal urate re-absorber expressed on the brush border membrane of proximal tubular cells. Among the already identified urate re-absorbers expressed on the apical side of the renal cells, URAT1 is most strongly associated with SUA levels in humans [7], as supported by the fact that *URAT1* is the causative gene for renal hypouricemia type 1 [12], an inherited disorder characterized by impaired urate re-absorption in the kidney that results in extremely low SUA levels (SUA ≤ 2 mg/dL; normal range: 3.0–7.0 mg/dL). With hyperuricemia patients, this transporter is also the pharmacological target of uricosuric agents, which promote the excretion of urate, such as benzbromarone [12], lesinurad [13], and dotinurad [14]. In this context, daily consumption of nutrients with URAT1-inhibitory activity may have a beneficial effect on SUA management in subjects with high SUA levels. Actually, food ingredients that inhibit URAT1 function have attracted great interest; we and other groups identified some such natural ingredients from fruit flavonoids [15], coumarins [16], and wood pigments [17]. Nevertheless, despite the nutritional significance of FAs, their effects on URAT1 activity remain to be elucidated.

In the present study, we examined the URAT1-inhibitory effects of 25 FAs using an in vitro transport assay with mammalian cells transiently expressing URAT1. The cell-based assay revealed that unsaturated FAs inhibited URAT1 more strongly than saturated FAs.

## 2. Materials and Methods

### 2.1. Materials

Critical materials and resources used in this study are summarized in Table 1. All other chemicals used were commercially available and of analytical grade. The FAs were re-dissolved with dimethyl sulfoxide (DMSO; Nacalai Tesque, Kyoto, Japan) after the solvents were gently evaporated with nitrogen gas on the heat block at 50 °C. All experiments were conducted with the same lot of each vector plasmid for URAT1 (URAT1 wild-type in pEGFP-C1) or mock (pEGFP-C1), which were derived from our previous study [15].

### 2.2. Cell Culture

Human embryonic kidney 293-derived 293A cells were maintained in Dulbecco’s modified Eagle’s medium (Nacalai Tesque, Kyoto, Japan) supplemented with 10% fetal bovine serum (Biowest, Nuaillé, France), 1% penicillin–streptomycin (Nacalai Tesque, Kyoto, Japan), 2 mM L-glutamine (Nacalai Tesque, Kyoto, Japan), and 1 × non-essential amino acid (Life Technologies, Carlsbad, CA, USA) at 37 °C in a humidified atmosphere of 5% (v/v) CO_2_ in air.

URAT1-expressing or mock plasmids were transfected into 293A cells using polyethylenimine “MAX” (PEI-MAX) (Polysciences, Warrington, PA, USA) as described previously [18], with some modifications. In brief, before transfection, 293A cells were seeded onto 12-well cell culture plates at a concentration of 0.92 × 10^5^ cells/cm^2^. Then, 24 h after seeding, each plasmid vector was transiently transfected into the cells using PEI-MAX (1 μg of plasmid/5 μL of PEI-MAX/well). The medium was replaced with fresh medium 24 h after transfection.

### 2.3. Preparation of Protein Lysates and Immunoblotting

Whole-cell lysates were prepared with cell lysis buffer A containing 50 mM Tris/HCl (pH 7.4), 1 mM dithiothreitol, 1% (*w/v*) Triton X-100, and protease inhibitor cOmplete, EDTA free (Roche, Basel, Switzerland), and were treated with peptide *N*-glycosidase F (PNGase F) (New England Biolabs, Ipswich, MA, USA) as described previously [19]. Protein concentration was determined using the Pierce^TM^ BCA Protein Assay Kit (Thermo Fisher Scientific, Kanagawa, Japan) with BSA as a standard, according to the manufacturer’s protocol.

Whole-cell lysate samples were separated by SDS-PAGE and transferred to an Immobilon-P PVDF membrane (Millipore, Bedford, MA, USA) by electroblotting at 15 V for 60 min, as described previously [15]. Blots were probed with appropriate antibodies (Table 1), and the signals were visualized by chemiluminescence and detected using a multi-imaging Analyzer Fusion Solo 4^TM^ system (Vilber Lourmat, Eberhardzell, Germany).

### 2.4. Confocal Microscopy

For confocal laser scanning microscopic observation, 48 h after the transfection, 293A cells were fixed with 4% paraformaldehyde for 15 min at room temperature, and further processed according to previous studies [15,20]. In brief, the cells were treated with a fluorescent wheat germ agglutinin conjugate (WGA, Alexa Fluor^®^ 594 conjugate; Thermo Fisher Scientific) to visualize plasma membranes, followed by nuclear staining using TO-PRO-3 Iodide (Molecular Probes, Eugene, OR, USA). Then, the cells were mounted in VECTASHIELD Mounting Medium (Vector Laboratories, Burlingame, CA, USA). To analyze the localization of EGFP-fused URAT1 protein, fluorescence was observed using the FV10i Confocal Laser Scanning Microscope (Olympus, Tokyo, Japan).

### 2.5. Urate Uptake Assay Using URAT1-Expressing 293A Cells

The urate uptake assay using URAT1-expressing 293A cells was conducted according to our previous studies [15,18] with minor modifications. In brief, 48 h after plasmid transfection, cells were washed twice with Cl^−^-free transport buffer (Buffer T2: 125 mM Na-gluconate, 4.8 mM K-gluconate, 1.2 mM KH_2_PO_4_, 1.2 mM MgSO_4_, 1.3 mM Ca-gluconate, 25 mM HEPES, 5.6 mM D-glucose, and pH 7.4) and pre-incubated in Buffer T2 for 15 min at 37 °C. The buffer was then exchanged with pre-warmed fresh Buffer T2 containing 5 μM [8-^14^C]-urate with or without test compound at the indicated concentrations (0, 0.1, 0.3, 1, 3, 10, 30, 100, or 300 μM), and the cells were further incubated for 20 sec; 1% DMSO was used as a vehicle control. The cells were subsequently washed five times with ice-cold Buffer T2 and then lysed with 500 μL of 0.2 M NaOH on ice with gentle shaking for 1 h. The lysates were neutralized with 100 μL of 1 M HCl. Then, the radioactivity in the lysate was measured using a liquid scintillator (Tri-Carb 3110TR; PerkinElmer, Waltham, MA, USA). The protein concentration was determined using the Pierce^TM^ BCA Protein Assay Kit. The urate transport activity was calculated as the incorporated clearance (μL/mg protein/min): (incorporated level of urate [disintegrations per minute (DPM)/mg protein/min]/urate level in the incubation mixture [DPM/μL]). URAT1-dependent urate transport activity was calculated by subtracting the urate transport activity of mock cells from that of URAT1-expressing cells.

Urate uptake was measured in the presence of several concentrations of each test compound to address their half maximal inhibitory concentration (IC_50_) values. URAT1-mediated transport activities were then expressed as a percentage of control (100%). Based on the calculated values, fitting curves were obtained according to the following formula using the least-squares method with Excel 2019 (Microsoft, Redmond, WA, USA):

(1) Predicted value %=100−Emax×CnEC50n+Cn

where E_max_ is the maximum effect, EC_50_ is the half maximal effective concentration, C is the concentration of the test compound, and n is the sigmoid-fit factor. Finally, based on the results, the IC_50_ was calculated as described previously [15].

### 2.6. Quantification and Statistical Analysis

All statistical analyses were performed using Excel 2019 with Statcel4 add-in software (OMS publishing, Saitama, Japan). Different statistical tests were used for different experiments as described in the figure legends, which include the number of biological replicates (*n*). Briefly, when analyzing multiple groups, the similarity of variance between groups was compared using Bartlett’s test. When passing the test for homogeneity of variance, a parametric Tukey–Kramer multiple-comparison test for all pairwise comparisons was used. To investigate the inhibitory effect of each FA on URAT1 function (vs. vehicle control indicated as 100%) in the screening stage, one-sample *t*-test (one-sided) was conducted. Statistical significance was defined as *p* < 0.05 or 0.01.

Each experiment was designed to use samples required to obtain informative results and sufficient material for subsequent studies. No specific statistical test was used to pre-determine the sample sizes empirically determined in the current study. All experiments were monitored in a non-blinded fashion.

## 3. Results

### 3.1. URAT1-Mediated Urate Uptake in 293A Cells

**Figure 1:**
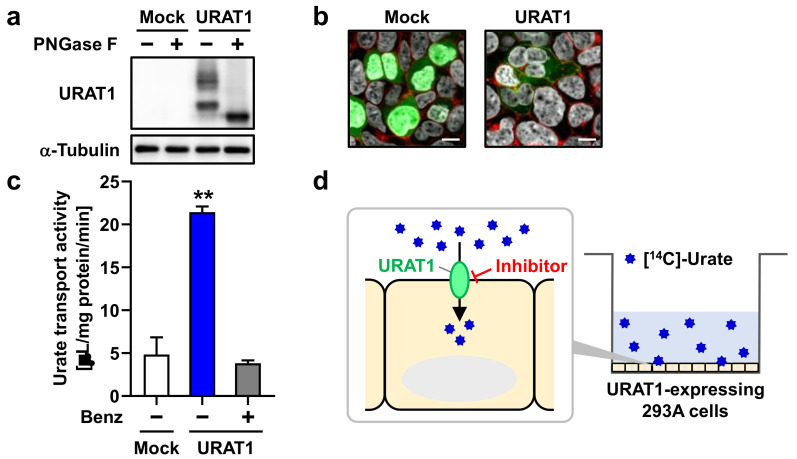
Cell-based urate transport assay with 293A cells transiently expressing URAT1. (**a**) Immunoblot detection of URAT1 protein in whole-cell lysates prepared 48 h after the transfection. α-Tubulin, a loading control. (**b**) Intracellular localization of URAT1. Confocal microscopy images were obtained 48 h after the transfection. Nuclei were stained with TO-PRO-3 iodide (gray); plasma membrane was labeled with Alexa Fluor^®^ 594-conjugated wheat germ agglutinin (red). Bars, 10 μm. (**c**) Urate transport activities. Urate uptake into cells treated with or without 30 μM of benzbromarone (Benz) was measured. Data are expressed as the mean ± SD; *n* = 3. **, *p* < 0.01 (Tukey–Kramer multiple-comparison test). (**d**) Schematic illustration of URAT1-mediated urate transport examined using 293A cells transiently expressing URAT1.

Prior to screening the inhibitory effects of 25 FAs on URAT1, we verified our cell-based assay system—an in vitro urate transport assay with mammalian cells transiently expressing URAT1 (Figure 1). Expression of EGFP-tagged URAT1 (EGFP-URAT1) as a matured N-linked glycoprotein (Figure 1a) and its plasma membrane localization (Figure 1b) in 293A cells were confirmed 48 h after plasmid transfection by immunoblotting and confocal microscopy, respectively. Next, we successfully detected URAT1-mediated urate uptake into URAT1-expressing cells, which showed a much stronger transport activity compared to mock cells, representing background urate uptake, indicating that the assay was suitable for the screening (Figure 1c). As expected, URAT1-mediated urate uptake was almost completely inhibited by benzbromarone (30 μM), a URAT1 inhibitor employed as a uricosuric drug. These results were consistent with our previous study [15]. A schematic illustration of this urate transport assay is shown in Figure 1d.

### 3.2. Unsaturated Fatty Acids Are Stronger Inhibitors of URAT1 Activity Than Saturated Fatty Acids

**Figure 2:**
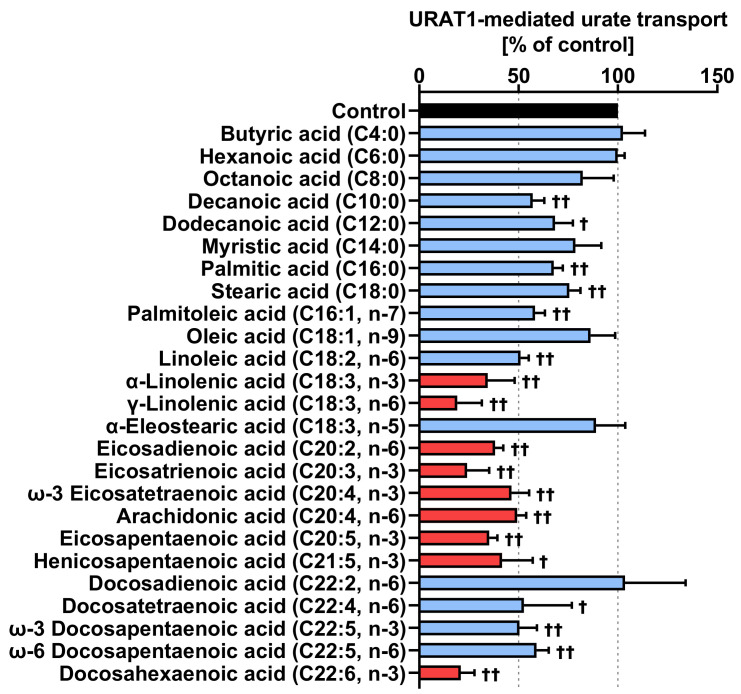
Inhibitory effects of each fatty acid on URAT1-mediated urate transport. The effects of each fatty acid (100 μM) on URAT1-mediated urate transport were investigated with the urate uptake assay. Control, vehicle (non-fatty acid treated) control. Data are expressed as the mean ± SD; *n* = 3–4. †, *p* < 0.05; ††, *p* < 0.01 vs. control (one-sample *t*-test). Red bars mean that the tested fatty acids inhibited URAT1-mediated urate transporter activity by over 50% compared to control.

**Figure A1:**
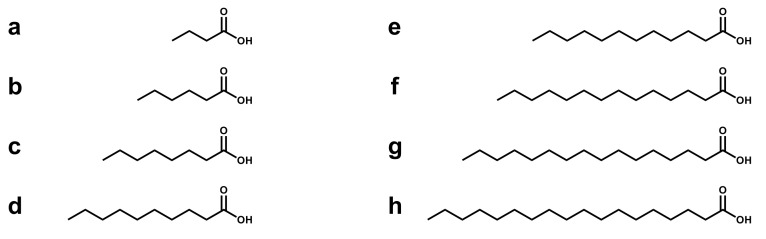
Chemical structures of saturated fatty acids tested in this study. (**a**) Butyric acid; (**b**) hexanoic acid; (**c**) octanoic acid; (**d**) decanoic acid; (**e**) dodecanoic acid; (**f**) myristic acid; (**g**) palmitic acid; (**h**) stearic acid.

**Figure A2:**
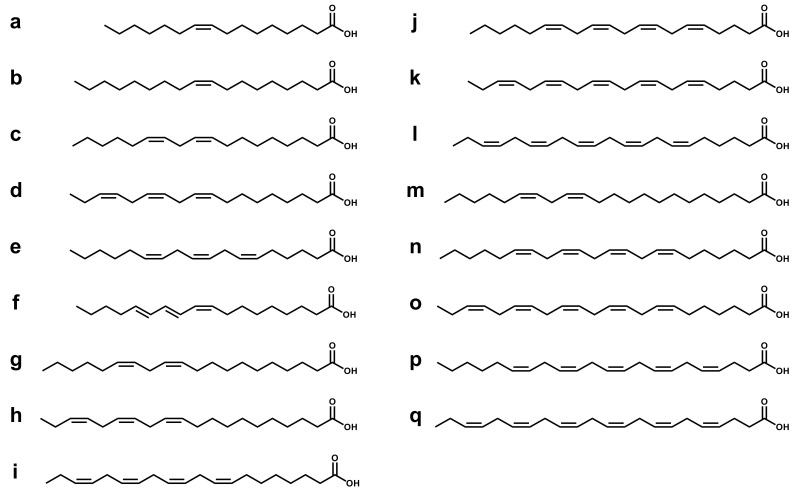
Chemical structures of unsaturated fatty acids tested in this study. (**a**) Palmitoleic acid; (**b**) oleic acid; (**c**) linoleic acid; (**d**) α-linolenic acid (ALA); (**e**) γ-linolenic acid; (**f**) α-eleostearic acid; (**g**) eicosadienoic acid; (**h**) eicosatrienoic acid; (**i**) ω-3 eicosatetraenoic acid; (**j**) arachidonic acid (ω-6 eicosatetraenoic acid); (**k**) eicosapentaenoic acid (EPA); (**l**) henicosapentaenoic acid; (**m**) docosadienoic acid; (**n**) docosatetraenoic acid; (**o**) ω-3 docosapentaenoic acid; (**p**) ω-6 docosapentaenoic acid; (**q**) docosahexaenoic acid (DHA).

Next, we examined the inhibitory effects of 25 FAs—8 saturated (Figure A1) and 17 unsaturated (Figure A2) FAs—at a concentration of 100 μM on URAT1 function (Figure 2). Despite some exceptions, in this study, almost all of the unsaturated FAs showed a stronger inhibitory effect on URAT1 than saturated FAs. Among the eight saturated FAs, relatively short FAs with chain lengths ranging from C4 to C8 had little effect on URAT1-mediated urate transport; instead, the others (C10 to C18) mildly inhibited URAT1 at the screening concentration. This result suggested that the length of FA could have a substantial effect on the URAT1-inhibitory activity of FAs. Among the 17 unsaturated FAs, 9 inhibited URAT1 activity by over 50%. We therefore focused on these candidates.

### 3.3. ω-3 Fatty Acids Are the Most Effective URAT1 Inhibitors

**Figure 3:**
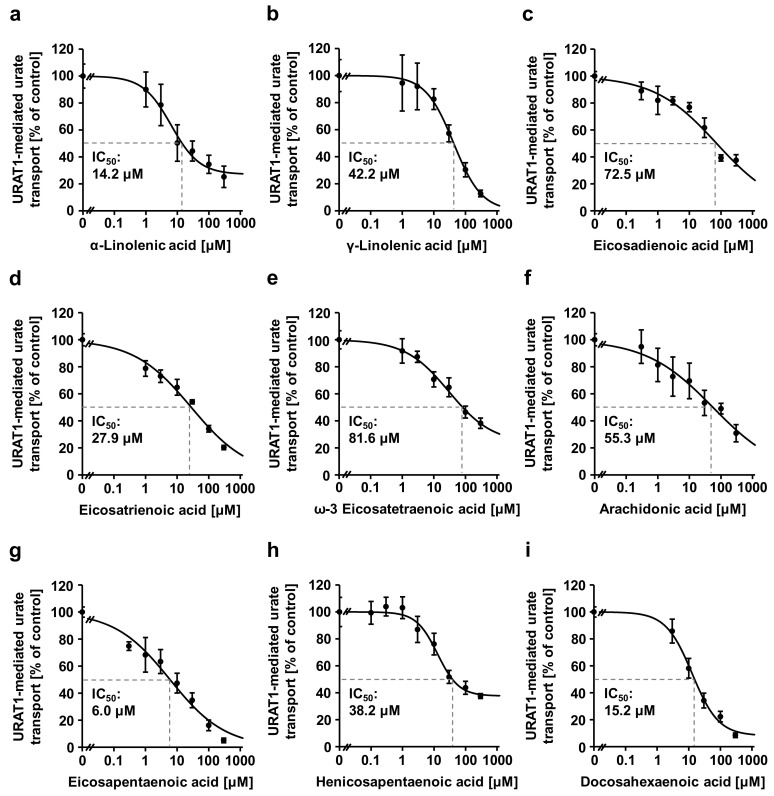
Concentration-dependent inhibition of URAT1-mediated urate transport by unsaturated fatty acids. The effects of each unsaturated fatty acid (0, 0.1, 0.3, 1, 3, 10, 30, 100, or 300 μM) on URAT1-mediated urate transport were investigated with the urate uptake assay. (**a**) α-Linolenic acid (ALA); (**b**) γ-linolenic acid; (**c**) eicosadienoic acid; (**d**) eicosatrienoic acid; (**e**) ω-3 eicosatetraenoic acid; (**f**) arachidonic acid (ω-6 eicosatetraenoic acid); (**g**) eicosapentaenoic acid (EPA); (**h**) henicosapentaenoic acid; (**i**) docosahexaenoic acid (DHA). Data are expressed as the mean ± SD; *n* = 4.

**Figure A3:**
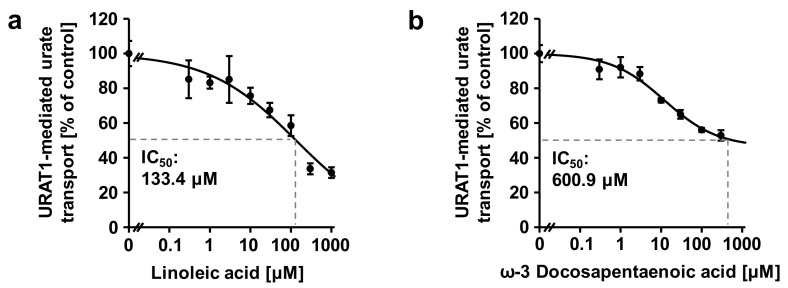
Concentration-dependent inhibition of URAT1-mediated urate transport by each fatty acid. Data are expressed as the mean ± SD; *n* = 4. (**a**) Linoleic acid; (**b**) ω-3 docosapentaenoic acid.

**Figure A4:**
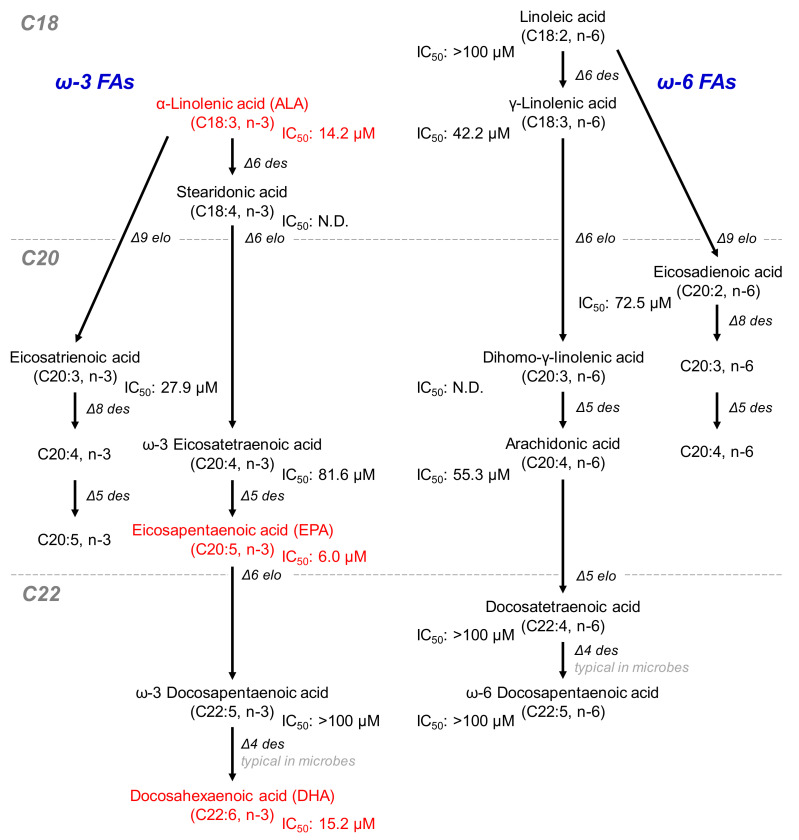
Biosynthetic route of fatty acids and half maximal inhibitory concentration (IC_50_) values of each tested fatty acid against the urate transport mediated by URAT1. The IC_50_ values are from Figure 2, Figure 3 and Figure A3. Three polyunsaturated fatty acids with substantial URAT1-inhibitory activities are indicated in red. N.D., not determined in this study; FAs, fatty acids; *des*, desaturase; *elo*, elongase. The metabolic pathway is adapted from a previous report [4], with some modifications.

Further investigation of the dose-dependent inhibitory effects of the nine unsaturated FAs on URAT1 determined the IC_50_ values that are illustrated in Figure 3. Based on the IC_50_ values, EPA was the strongest URAT1 inhibitor among the nine unsaturated FAs examined. Furthermore, EPA inhibited URAT1 activity more strongly than the other unsaturated FAs at low concentrations (≤1 μM) (Figure 3g). Second to EPA, its biosynthetic precursor α-linolenic acid (ALA) (Figure 3a) as well as its product DHA (Figure 3i) strongly inhibited URAT1, while ω-3 docosapentaenoic acid (DPA) showed an IC_50_ of > 100 μM (Figure 2 and Figure A3). Additionally, contrary to ALA, linoleic acid (LA) had a high IC_50_ (133 μM); however, LA could inhibit URAT1 at low concentrations (≤1 μM) (Figure A3). Interestingly, considering the biosynthetic pathways of the above-described FAs distinguished by their structural feature (ω-3 or ω-6 family) (Figure A4), ω-3 FAs seem to inhibit URAT1 more effectively than ω-6 FAs. Given that elevated intake of ω-6 FAs may reportedly promote inflammation, while ω-3 FAs help reduce it [21], ω-3 FAs will be preferable to ω-6 FAs for the prevention of hyperuricemia/gout.

## 4. Discussion

In this study, to the best of our knowledge, we revealed for the first time the inhibitory effects of FAs on URAT1-dependent urate transport in vitro. While further studies are highly warranted to address the pathophysiological impact of our findings in terms of the possible effect on SUA and renal urate excretion in hyperuricemia model animals as well as in humans, the obtained results will open a new avenue for FAs as nutritionally important substances influencing human health.

Our findings may extend the potentially beneficial effects of PUFAs of the ω-3 family on reducing the risk of hyperuricemia/gout. In fact, recent studies have shown that a dietary intake of certain ω-3 PUFAs, such as EPA and DHA can, at least partly, reduce a wide number of inflammation-related biological reactions [1,4,5,22]. With respect to gout, a small case–control study on patients with this inflammatory condition showed an association between high ω-3 FA levels in the blood and lower frequency of gout attacks [23]. Additionally, ω-3 PUFA-rich fish consumption was reportedly associated with lower risk of recurrent gout attacks in a case-crossover study [24]. Moreover, the anti-inflammatory effects of ω-3 PUFAs were supported by the results obtained in animal models of acute inflammation induced by monosodium urate crystals [25,26]. Thus, in addition to the anti-inflammatory effects previously observed, it should be confirmed in future studies whether ω-3 PUFAs can exhibit uricosuric effects in hyperuricemia models or not.

The potential effects of the daily consumption of ω-3 PUFA-enriched foods on SUA will also be of interest. While the available information is limited and currently inconclusive, a randomized controlled trial in young healthy subjects showed that daily intake of fish oil (2 g; majorly consisting of DHA and EPA) resulted in a significant decrease of SUA after 4 and 8 weeks of supplementation [27]. A similar significant decline in SUA was also observed in healthy elderly men consuming daily supplement pills characterized by ω-3 FAs such as DHA and EPA for three months, although the change was not extensive [28]. Based on these pieces of evidence, increasing the daily intake of ω-3 FAs via eating pattern changes, such as appropriate choice of aliments and cooking oils, might be beneficial to health in terms of SUA management. On the other hand, the behavior of ω-3 FAs in the body has been hardly investigated in those clinical investigations, which warrants further studies focusing on the beneficial effects of dietary and/or endogenously produced ω-3 FAs on the renal urate handling in the body. Additionally, in such cases, not only the URAT1-inhibitory activity but also the disposition of target FAs should be considered.

There were some limitations to our study. First, the present study could not reveal how the FAs inhibited URAT1 function. Addressing this issue in the future will provide a deeper insight into the latent mechanistic features of URAT1. As an antiporter, URAT1 mediates urate transport in exchange for monocarboxylates such as lactate [12], which suggests that URAT1 must have at least two substrate recognition sites in its protein structure. Considering that FAs are carboxylic acids with a long aliphatic chain, they might affect the recognition and/or subsequent membrane transport of the counterpart substrates by URAT1 rather than the recognition of urate. Second, we could not exclude the possibility that the FAs affected the plasma membrane properties, which might result in the indirect decrease of URAT1 function. Nonetheless, given that the interaction of free FAs with cellular membranes occurs within minutes [21] and usually requires biochemical conversion of FAs into phospholipids, the experimental period we used in this study, 20 s incubation for urate uptake, was so short that the tested FAs must have had a negligible effect on the plasma membranes during the assay. Finally, the effects of FAs on other physiologically important urate transporters—GLUT9/SLC2A9 [29,30], OAT10/SLC22A13 [31], and ABCG2/BCRP [32,33,34]—remain to be elucidated. Since such urate transporters, including URAT1, coordinately regulate the behavior of urate in the human body, comprehensive understanding of the latent interaction between FAs and these transporters should be addressed in the future. Among them, GLUT9 expressed on the basal membrane of proximal tubular cells is involved in the urate transport from the cells to the blood as a counterpart of URAT1 [7]; *GLUT9* is the causative gene for renal hypouricemia type 2 [30]. Given these pieces of information, GLUT9 has the highest priority in the future investigation.

## 5. Conclusions

In conclusion, we herein found that FAs, especially ω-3 PUFAs such as EPA and DHA, could inhibit URAT1. To gain insight into the potential SUA-lowering effects of certain FAs, further investigations including human studies are required.

## Figures and Tables

**Table 1:** Key resources.

Reagent or Resource	Source	Identifier
***Antibodies***		
Rabbit polyclonal anti-EGFP	Life Technologies	Cat# A11122; RRID: AB_221569; 1:1,000 dilution ^1^
Rabbit polyclonal anti-α-tubulin	Abcam	Cat# ab15246; RRID: AB_301787; 1:1,000 dilution ^1^
Donkey anti-rabbit IgG-horseradish peroxidase (HRP)-conjugate	GE Healthcare	Cat# NA934V; RRID: AB_772206; 1:3,000 dilution ^1^
***Chemicals***		
[8-^14^C]-Uric acid (53 mCi/mmol)	American Radiolabeled Chemicals	Cat# ARC0513
Arachidonic acid	Cayman Chemical	Cat# 90010; CAS: 506-32-1; Purity: ≥98%
Benzbromarone	FUJIFILM Wako Pure Chemical	Cat# 028-1585; CAS: 3562-84-3; Purity: >98%
Butyric acid	SIGMA-ALDRICH	Cat# B103500-5ML; CAS: 107-92-6; Purity: ≥99%
Decanoic acid	FUJIFILM Wako Pure Chemical	Cat# 033-01073; CAS: 334-48-5; Purity: ≥98%
Dimethyl sulfoxide	Nacalai Tesque	Cat# 13445-74; CAS: 67-68-5
Docosadienoic acid	Cayman Chemical	Cat# 20749; CAS: 17735-98-7; Purity: ≥98%
Docosahexaenoic acid	Cayman Chemical	Cat# 90310; CAS: 6217-54-5; Purity: ≥98%
Docosatetraenoic acid	Cayman Chemical	Cat# 90300; CAS: 28874-58-0; Purity: ≥98%
Dodecanoic acid	SIGMA-ALDRICH	Cat# L556-25G; CAS: 143-07-7; Purity: ≥98%
Eicosadienoic acid	Cayman Chemical	Cat# 90330; CAS: 2091-39-6; Purity: ≥98%
Eicosapentaenoic acid	Cayman Chemical	Cat# 90110; CAS: 10417-94-4; Purity: ≥98%
Eicosatrienoic acid	Cayman Chemical	Cat# 90192; CAS: 20590-32-3; Purity: ≥98%
Henicosapentaenoic acid	Cayman Chemical	Cat# 10670; CAS: 24257-10-1; Purity: ≥95%
Hexanoic acid	FUJIFILM Wako Pure Chemical	Cat# 081-06292; CAS: 142-62-1; Purity: ≥99%
Linoleic acid	Cayman Chemical	Cat# 90150; CAS: 60-33-3; Purity: ≥98%
Myristic acid	FUJIFILM Wako Pure Chemical	Cat# 130-03432; CAS: 544-63-8; Purity: ≥98%
Octanoic acid	SIGMA-ALDRICH	Cat# C2875-10ML; CAS: 124-07-2; Purity: ≥99%
Oleic acid	Cayman Chemical	Cat# 90260; CAS: 112-80-1; Purity: ≥98%
Palmitic acid	Cayman Chemical	Cat# 10006627; CAS: 57-10-3; Purity: ≥98%
Palmitoleic acid	Cayman Chemical	Cat# 10009871; CAS: 373-49-9; Purity: ≥99%
Polyethelenimine “MAX”	Polysciences	Cat# 24765; CAS: 49553-93-7
Stearic acid	SIGMA-ALDRICH	Cat# S4751-1G; CAS: 57-11-4; Purity: ≥98.5%
α-Eleostearic acid	Cayman Chemical	Cat# 10008349; CAS: 506-23-0; Purity: ≥95%
α-Linolenic acid	Cayman Chemical	Cat# 90210; CAS: 463-40-1; Purity: ≥98%
γ-Linolenic acid	Cayman Chemical	Cat# 90220; CAS: 506-26-3; Purity: ≥98%
ω-3 Eicosatetraenoic acid	Larodan Fine Chemicals	Cat# 10-2024; CAS: 24880-40-8; Purity: ≥98%
ω-3 Docosapentaenoic acid	Cayman Chemical	Cat# 90165; CAS: 24880-45-3; Purity: ≥98%
***Critical Commercial Assays***		
Pierce^TM^ BCA Protein Assay Reagent A & B	Thermo Fisher Scientific	Cat# 23223, Cat# 23224
PureLink^TM^ HiPure Plasmid Filter Midiprep Kit	Thermo Fisher Scientific	Cat# K210015
***Recombinant DNA***		
The complete URAT1 cDNA	Miyata et al., 2016 [18]	NCBI Reference Sequence: NM_144585.3
***Experimental Models: Cell Lines***		
293A	Invitrogen	R70507
***Software and Algorithms***		
Excel 2019	Microsoft	https://products.office.com/ja-jp/home
Statcel4 add-in software	OMS Publishing	http://www.oms-publ.co.jp/

^1^ All antibodies were used at indicated dilutions in Tris-buffered saline containing 0.05% Tween 20 and 1% bovine serum albumin for 1 h at room temperature.
